# Antidote to Strychnia

**Published:** 1876-08

**Authors:** 


Antidote to Strychnia.—The East Indian physicians recommend nicotia as the surest antidote, which is given in exceedingly small quantities in sherry several times a day. In default of nicotia, the decoction of tobacco leaves (½ ounce to a pint) is given.—*American Journal of Pharmacy.*

